# Can Short and Partial Observations Reduce Model Error and Facilitate Machine Learning Prediction?

**DOI:** 10.3390/e22101075

**Published:** 2020-09-24

**Authors:** Nan Chen

**Affiliations:** Department of Mathematics, University of Wisconsin-Madison, 480 Lincoln Dr. Madison, Madison, WI 53706, USA; chennan@math.wisc.edu

**Keywords:** model error, short and partial observations, data assimilation, conditional sampling, machine learning, non-Gaussian systems

## Abstract

Predicting complex nonlinear turbulent dynamical systems is an important and practical topic. However, due to the lack of a complete understanding of nature, the ubiquitous model error may greatly affect the prediction performance. Machine learning algorithms can overcome the model error, but they are often impeded by inadequate and partial observations in predicting nature. In this article, an efficient and dynamically consistent conditional sampling algorithm is developed, which incorporates the conditional path-wise temporal dependence into a two-step forward-backward data assimilation procedure to sample multiple distinct nonlinear time series conditioned on short and partial observations using an imperfect model. The resulting sampled trajectories succeed in reducing the model error and greatly enrich the training data set for machine learning forecasts. For a rich class of nonlinear and non-Gaussian systems, the conditional sampling is carried out by solving a simple stochastic differential equation, which is computationally efficient and accurate. The sampling algorithm is applied to create massive training data of multiscale compressible shallow water flows from highly nonlinear and indirect observations. The resulting machine learning prediction significantly outweighs the imperfect model forecast. The sampling algorithm also facilitates the machine learning forecast of a highly non-Gaussian climate phenomenon using extremely short observations.

## 1. Introduction

Predicting complex nonlinear turbulent dynamical systems is an important and practical topic. In many areas including engineering, geophysics, and climate science, dynamical or statistical models are widely used for prediction. However, due to the lack of a complete understanding of nature, the ubiquitous model error may greatly affect the prediction performance. On the other hand, machine learning forecasts based on the properties learned from massive training data possess a large potential to overcome the model error and they have become the predominant forecast methods in quite a few industrial and engineering problems [1,2,3]. However, the prediction of many climate and geophysical phenomena using machine learning algorithms can still be very challenging. This is because the high-resolution satellites and other refined measurements were not widely developed until recent times. As a result, the available training data are very limited with about only 40 years, which is far from enough to train the machine learning algorithms for predicting interannual or decadal variability. Another fundamental difficulty in utilizing machine learning to predict nature is that only partial observations are available in most applications. In other words, the typical observations involve merely a small subset of the variables and sometimes there is even no direct observations for any variables of interest. Reconstructing the training data of the unobserved variables is quite difficult without the aid of a suitable model.

Since the data in climate science, geophysics, and many other complex nonlinear systems are spatiotemporally correlated and intrinsically chaotic, the traditional data augmentation methods [4,5,6] for static data are not applicable to expanding the training data set of these problems. On the other hand, thanks to the development of many physics-based dynamical models in describing nature, long correlated time series from these models have been used for training the machine learning algorithms [7,8]. The resulting machine learning prediction can often outperform the simple model forecast. However, such an enhancement of prediction accuracy mainly comes from the improvement of the forecast methodology whereas model error remains in the training data. Thus, incorporating the available short observations into the imperfect model is essential to improving the quality of the training data.

Data assimilation is a well-known technique that combines observations with an imperfect model to improve the statistical state estimation [9,10,11,12]. The resulting posterior distribution is typically more informative and accurate than the model’s prior distribution. Data assimilation also plays an important role in recovering the states of the unobserved variables via their dynamical coupling with the observed ones in the model.

Despite the idea of combining partial observations with an imperfect model to improve the quality of the training data for machine learning, there remain two key issues to be resolved. First, data assimilation only provides a statistical state estimation at each time instant. The more desired format of training data are the time series that describes the spatiotemporal evolution of the underlying dynamics. In practice, the time series by collecting all the posterior mean estimates is often used as an approximation of the true signal [13]. However, the posterior mean time series can be biased in reproducing even the basic dynamical properties of nature (See Appendix A). Second, since the length of the assimilated states is consistent with that of the short observations, the insufficient training data remain as an unsolved problem.

In this article, an efficient and dynamically consistent conditional sampling algorithm for nonlinear time series is developed that resolves the two issues discussed above. The sampling algorithm starts with a forward and a backward data assimilation procedure to characterize the correct uncertainty of the posterior estimates. Then, the path-wise temporal dependence conditioned on the observations is combined with the point-wise posterior estimates to develop a recursive sampling scheme. For a rich class of nonlinear and non-Gaussian systems, the conditional sampling is carried out by solving a simple stochastic differential equation (SDE), where the short and partial observations serve as the implicit input. This allows an extremely efficient way to sample the nonlinear time series even in high dimensions. One crucial feature of the resulting sampled trajectories is that they outweigh the posterior mean time series in that they succeed in reproducing the exact dynamical and statistical characteristics of nature in the perfect model setup. In the presence of an imperfect model, the model error can be significantly mitigated in the sampled trajectories due to the extra information from observations. What remains is to cope with the short observations. Here, the posterior uncertainties play a vital role in generating multiple distinct sampled trajectories conditioned on the same short observations, which effectively provide a large training set. In addition, this conditional sampling technique allows for sampling the trajectories of the unobserved variables. All these features facilitate the machine learning training and advance the effective prediction.

The rest of the article is organized as follows. The efficient and dynamically consistent conditional sampling algorithm is developed in Section 2. The prediction schemes are described in Section 3. Section 4 aims at improving the prediction of multiscale compressible shallow water flows using indirect observations. Section 5 focuses on advancing the forecast of a highly non-Gaussian climate phenomenon using extremely short observations. The article is concluded in Section 6.

## 2. The Nonlinear Models and the Conditional Sampling Algorithm

The focus here is on a rich class of high-dimensional nonlinear and non-Gaussian turbulent models, the structure of which allows using closed analytic formulae to develop an efficient and dynamically consistent conditional sampling algorithm. The procedure of deriving such a conditional sampling technique can be extended to general nonlinear systems using particle methods.

### 2.1. The Nonlinear Modeling Framework

Consider the following class of nonlinear and non-Gaussian systems [14,15],

(1a) $$\begin{array}{cc} \;dX(t) & =\left[A_{0}\left(X,t\right)+A_{1}\left(X,t\right)Y\left(t\right)\right]\;dt+B_{1}\left(X,t\right)\;dW_{1}\left(t\right)+B_{2}\left(X,t\right)\;dW_{2}\left(t\right), \end{array}$$

(1b) $$\begin{array}{cc} \;dY(t) & =\left[a_{0}\left(X,t\right)+a_{1}\left(X,t\right)Y\left(t\right)\right]\;dt+b_{1}\left(X,t\right)\;dW_{1}\left(t\right)+b_{2}\left(X,t\right)\;dW_{2}\left(t\right), \end{array}$$

where X and Y are both multi-dimensional state variables. In (1), $A_{0}$, $A_{1}$, $a_{0}$, $a_{1}$, $b_{1}$, $b_{2}$, $B_{1}$ and $B_{2}$ are vectors and matrices that depend nonlinearly on the state variables X and time *t*, while $W_{1}$ and $W_{2}$ are independent white noise. For nonlinear systems with partial observations, X can be regarded as the collection of the observed variables while Y contains the variables that are not directly observed.

The systems in (1) are called conditional Gaussian nonlinear systems due to the fact that conditioned on a given realization of X(s) for s≤t, the distribution of Y(t) is Gaussian. Despite the conditional Gaussianity, both the joint and marginal distributions can be highly non-Gaussian. Extreme events, intermittency, and complex nonlinear interactions between different variables all appear in such systems. The framework includes many physics-constrained nonlinear stochastic models, large-scale dynamical models in turbulence, fluids, and geophysical flows, as well as stochastically coupled reaction–diffusion models in neuroscience and ecology. A gallery of examples of conditional Gaussian systems in engineering, neuroscience, ecology, fluids, and geophysical flows can be found in [15]. Some well-known dynamical systems that belong to this framework are the noisy versions of various Lorenz systems, a variety of the stochastically coupled FitzHugh–Nagumo model, and the Boussinesq equations with noise.

### 2.2. The Nonlinear Data Assimilation

Data assimilation or filtering aims at solving the conditional (or posterior) distribution p(Y(t)|X(s),s≤t). One advantage of the conditional Gaussian nonlinear systems (1) is that such a conditional distribution can be written down using closed analytic formulae.

**Theorem** **1** (Nonlinear Optimal Filter [14])**.** *For the conditional Gaussian nonlinear systems (1), given one realization of X(s) for s∈[0,t], the conditional distribution $p\left(Y\left(t\right)|X\left(s\right),s\leq t\right)∼N\left(\mu_{f}\left(t\right),R_{f}\left(t\right)\right)$ is Gaussian. The conditional mean $\mu_{f}$ and the conditional covariance $R_{f}$ are given by the following explicit formulae:*

(2a) $$\begin{array}{cc} \;d\mu_{f} & =\left(a_{0}+a_{1}\mu_{f}\right)\;dt+\left(b\circ B+R_{f}A_{1}^{*}\right){\left(B\circ B^{*}\right)}^{-1}\left(\;dX-\left(A_{0}+A_{1}\mu_{f}\right)\;dt\right), \end{array}$$

(2b) $$\begin{array}{cc} \;dR_{f} & =\left(a_{1}R_{f}+R_{f}a_{1}^{*}+b\circ b-\left(b\circ B+R_{f}A_{1}^{*}\right){\left(B\circ B\right)}^{-1}\left(B\circ b+A_{1}R_{f}\right)\right)\;dt, \end{array}$$

where $b\circ b=b_{1}b_{1}^{*}+b_{2}b_{2}^{*}$, $B\circ B=B_{1}B_{1}^{*}+B_{2}B_{2}^{*}$ and $b\circ B=b_{1}B_{1}^{*}+b_{2}B_{2}^{*}$.

Here, the “optimality” is in the Bayesian sense. From now on, we assume b∘B=0, which is the case in most applications.

### 2.3. The Optimal Conditional Sampling

The data assimilation exploits the observational information up to the current time instant for improving the initialization of real-time prediction. On the other hand, the optimal offline point-wise statistical state estimation can be carried out by making use of the observational information in the entire training period. This leads to a more accurate state estimation and is achieved by a forward pass of filtering and a backward pass of smoothing.

**Theorem** **2** (Nonlinear Optimal Smoother [16])**.** *For the conditional Gaussian nonlinear systems (1), given one realization of X(t) for t∈[0,T], the optimal smoother estimate $p\left(Y\left(t\right)|X\left(s\right),s\in \left[0,T\right]\right)∼N\left(\mu_{s}\left(t\right),R_{s}\left(t\right)\right)$ is conditional Gaussian, where the conditional mean $\mu_{s}\left(t\right)$ and the conditional covariance $R_{s}\left(t\right)$ satisfy the following equations:*

(3a) $$\begin{array}{cc} \overset{\leftarrow }{\;d\mu_{s}} & =\left(-a_{0}-a_{1}\mu_{s}+\left(b\circ b\right)R_{f}^{-1}\left(\mu_{f}-\mu_{s}\right)\right)\;dt, \end{array}$$

(3b) $$\begin{array}{cc} \overset{\leftarrow }{\;dR_{s}} & =-\left(\left(a_{1}+\left(b\circ b\right)R_{f}^{-1}\right)R_{s}+R_{s}\left(a_{1}^{*}+\left(b\circ b\right)R_{f}^{-1}\right)-b\circ b\right)\;dt. \end{array}$$

*The backward notations on the left-hand side of (3) are understood as $\overset{\leftarrow }{\;d\mu_{s}}=\lim_{\Delta t\to 0}\mu_{s}\left(t\right)-\mu_{s}\left(t+\Delta t\right)$. The starting value of the nonlinear smoother $\left(\mu_{s}\left(T\right),R_{s}\left(T\right)\right)$ is the same as the filter estimate at the endpoint $\left(\mu_{f}\left(T\right),R_{f}\left(T\right)\right)$.*

The nonlinear smoother provides the optimal statistical state estimate at each time instant *t*. However, merely using the information from the smoother posterior distributions (3) is not sufficient to draw unbiased model trajectories conditioned on the observations. This is because, in addition to the point-wise statistical state estimates (namely at each fixed time instant) from the smoother (3), the conditional temporal dependence at nearby time instants also needs to be taken into account in creating these conditional sampled trajectories. In practice, the posterior mean time series is often used as a surrogate of the recovered model trajectory conditioned on the observations. However, the posterior uncertainty and its temporal correlation are completely ignored in such an approximation. The consequence is that the posterior mean time series fails to capture many key dynamical and statistical features of the underlying dynamics, such as the temporal autocorrelation function (ACF) and the PDF (See Appendix A). Note that a naive way of involving the posterior uncertainty in sampling hidden trajectories conditioned on the observations is to draw independent random numbers from the posterior distributions at different time instants and then connect them together. However, such an approach not only leads to a noisy time series but fails to capture the underlying dynamical features as well due to the lack of incorporating the temporal correlation of the uncertainty.

The result presented below exploits both the conditional path-wise temporal dependence and the point-wise optimal state estimate from the nonlinear smoother (3) to build an optimal conditional sampling algorithm. For the nonlinear systems (1), the conditional sampling can be carried out in an extremely efficient way by solving a simple SDE.

**Theorem** **3** (Conditional Sampling Formula)**.** *For the conditional Gaussian nonlinear systems (1), conditioned on one realization of X(t) for t∈[0,T], the optimal conditional sampling formula of the trajectories of Y(t) in the same time interval satisfies the following explicit formula:*

(4) $$\overset{\leftarrow }{\;dY}=\overset{\leftarrow }{\;d\mu_{s}}-\left(a_{1}+\left(b\circ b\right)R_{f}^{-1}\right)\left(Y-\mu_{s}\right)\;dt+{\left(b\circ b\right)}^{1/2}\;dW_{Y}.$$

*where $W_{Y}$ is an independent white noise source and the square root of the positive definite matrix b∘b is unique. The initial value of Y is drawn from $N(\mu_{f}\left(T\right),R_{f}\left(T\right))$.*

See Appendix D for the proof of this Theorem. The mathematical conciseness of (4) indicates that the sampled trajectories meander around the smoother mean time series. Meanwhile, the second term on the right-hand side indicates a correlated uncertainty in the sampled trajectories. Such a temporal correlated uncertainty plays a crucial role for the sampled trajectories to perfectly reproduce the path-wise and statistical features of nature in the absence of model error, which is however lacked in the posterior mean time series. The uncertainty also facilitates the sampling of multiple distinct trajectories conditioned on the same short observational time series, which effectively provide a large training set for the machine learning algorithms. Notably, in the presence of an imperfect model, the model error can be significantly mitigated in the sampled trajectories due to the extra information from observations. The machine learning prediction based on these sampled trajectories is thus expected to outperform the model forecast using the imperfect model.

Thanks to the explicit formula in (4), the conditional sampling procedure is computationally much cheaper than the traditional particle methods. In Appendix A, an alternative conditional sampling formula is included. It requires only the filter estimate, which further reduces the computational cost. In addition, a block decomposition technique [17] can be adopted here for efficiently sampling many high-dimensional systems.

**Figure 1:**
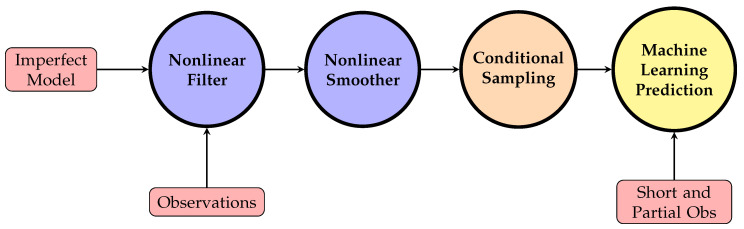
Illustration of the conditional sampling procedure and its application to the augmentation of the training data for machine learning forecasts.

Finally, Figure 1 shows a schematic illustration of the entire procedure. Here, the nonlinear smoother is crucial for the state estimation. This is because the smoother improves the filter estimates by exploiting the entire observational information and leads to an unbiased conditional state estimation. Utilizing the point-wise state estimates and the temporal dependence to derive the formula of the path-wise conditional sampling is another key step that fundamentally differs from the state estimation using data assimilation.

## 3. The Prediction Schemes

### 3.1. The Machine Learning Algorithm

The purpose of this article is to elucidate the advantage of using the multiple conditional sampled trajectories to reduce the model error and improve the machine learning prediction. To this end, designing sophisticated machine learning algorithms is not the main focus here. Throughout this article, the long short-term memory (LSTM) network [18] is adopted as the machine learning algorithm. The LSTM network is an artificial recurrent neural network architecture in deep learning. Unlike the standard feed forward neural networks, LSTM has feedback connections. Therefore, it is applicable to predicting sequential data, in particular the time series from complex nonlinear dynamical systems. Only the vanilla LSTM network is used here. It starts with a sequence input layer followed by an LSTM layer. The network ends with a fully connected layer and a regression output layer.

### 3.2. The Ensemble Forecast

The ensemble forecast is a widely used model-based prediction approach, which involves making a set of forecasts. Due to the intrinsic chaotic behavior of the underlying dynamics, different forecast trajectories are distinct from each other. The ensemble mean forecast time series is often regarded as the path-wise prediction of the true signal and the ensemble spread characterizes the forecast uncertainty. In the following tests, the ensemble forecast is adopted as the prediction scheme for both the perfect and imperfect models. The associated ensemble mean time series will be compared with the LSTM prediction.

## 4. Predicting Multiscale Compressible Shallow Water Flows Using Lagrangian Observations

In many applications, one fundamental difficulty is that the variables of interest are not directly observed. The goal of this section is to predict multiscale compressible shallow water flows, where the available indirect observations are the Lagrangian tracer trajectories that are transported by the underlying flow field [19,20,21]. To mimic the real-world scenario, the only information in hand is an imperfect flow model and a short observational trajectory of the Lagrangian tracers.

### 4.1. The Shallow Water Equation

The linear rotating shallow water equation with double periodic boundary conditions in ${\left[-\pi ,\pi \right)}^{2}$ domain reads [22,23]

(5) $$\begin{array}{cc} \;\;\frac{\partial u}{\partial t}+\epsilon^{-1}u^{⊥} & =-\epsilon^{-1}\nabla \eta ,\\ \;\;\frac{\partial \eta }{\partial t}+\epsilon^{-1}\delta \nabla \cdot u & =0, \end{array}$$

where u=(u,v) is the two-dimensional velocity field and η is the height function. The non-dimensional numbers are ϵ=Ro, $\delta ={\mathrm{Ro}}^{2}{\mathrm{Fr}}^{-2}$, where Ro is the Rossby number that represents the ratio between the Coriolis term and the advection term, and Fr is the Froude number. For most geophysical problems, ϵ ranges from O(0.1) to O(1), representing fast to moderate rotations while δ=1 is a typically choice. Applying a plane wave ansatz, the solution of (5) is given by [22]

(6) $$\left(\begin{array}{c} v(x,t)\\ \eta (x,t) \end{array}\right)=\sum_{k\in Z^{2},\zeta \in \left\{B,\pm \right\}}{\hat{u}}_{k,\zeta }\left(t\right)e^{ik\cdot x}r_{k,\zeta },$$

where $k=(k_{1},k_{2})$ is the two-dimensional Fourier wavenumber. The set {B,+,−} represents three different modes associated with each Fourier wavenumber. The modes with ζ=B are the geostrophically balanced (GB) modes, where the GB relation $u^{⊥}=-\nabla \eta$ always holds. The GB flows are incompressible, which is embodied in the eigenvector $r_{k,\zeta }$, and the associated phase speed is $\omega_{k,B}=0$. The modes with ζ=± represent the compressible gravity waves with the phase speeds $\omega_{k,\pm }=\pm \epsilon^{-1}\sqrt{{\left|k\right|}^{2}+1}$. See Appendix B for details. Here, the temporal evolution of each random Fourier coefficient ${\hat{u}}_{k,\zeta }\left(t\right)$ is governed by a linear Gaussian process [24],

(7a) $$\begin{array}{cc} \;d{\hat{u}}_{k,B} & =\left(-d_{k,B}{\hat{u}}_{k,B}+f_{k,B}\left(t\right)\right)\;dt+\sigma_{k,B}\;dW_{k,B}\left(t\right), \end{array}$$

(7b) $$\begin{array}{cc} \;d{\hat{u}}_{k,\pm } & =\left(\;\left(-d_{k,\pm }+i\omega_{k,\pm }\right){\hat{u}}_{k,\pm }+f_{k,\pm }\left(t\right)\;\right)\;dt+\sigma_{k,\pm }\;dW_{k,\pm }\left(t\right). \end{array}$$

Such an representation is widely used in practice [25,26,27], where the damping and random noise allow a simple way to mimic the turbulent features and the interactions between the resolved and unresolved scales. The large-scale deterministic forcings $f_{k,B}\left(t\right)$ and $f_{k,\pm }\left(t\right)$ represent for example the seasonal cycle. It is clear that, when ϵ is small, the model becomes a multiscale system with slow GB flows and fast gravity waves.

### 4.2. The Lagrangian Observations

Assume there are *L* tracers. The equation of each tracer trajectory $x_{l}=\left(x_{l},y_{l}\right)$ is given by the Newton’s law together with some small-scale uncertainties

(8) $$\;dx_{l}=v\left(x_{l}\right)\;dt+\sigma_{x}\;dW_{l}=\sum_{k\in Z^{2},\zeta \in \left\{B,\pm \right\}}{\hat{u}}_{k,\zeta }\left(t\right)e^{ik\cdot x_{l}}{\overset{˜}{r}}_{k,\zeta }+\sigma_{x}\;dW_{l},$$

where ${\overset{˜}{r}}_{k,\zeta }$ is a two-dimensional vector, containing the first two components of the eigenvector $r_{k,\zeta }$. It is important to note that the GB and the gravity modes are coupled in the observations. In addition, the tracer equation is highly nonlinear because the prognostic variable $x_{l}$ appears in the exponential function on the right-hand side. These features lead to a tough test in recovering and sampling the underlying velocity fields. Nevertheless, despite the intrinsic nonlinearity, the coupled system (6)–(8) belongs to the modeling framework (1), which facilitates the conditional sampling of the flow trajectories [28].

### 4.3. Setup of the Numerical Tests

The modes with wavenumbers $-1\leq k_{1},k_{2}\leq 1$ are used in the study here, which means the total number of the Fourier modes is 9×3=27. However, the first two components of the (0,0)-th GB mode are zero. This mode often represents the given background flow. Since it has no contribution to the observational process, it is removed here. Thus, the degree of freedom (DoF) of the underlying model is DoF =26. See Appendix B for details. The parameters in (7) are $d_{k,B}=d_{k,g}=0.5$, $\sigma_{k,B}=\sigma_{k,\pm }=0.4$ and $f_{k,B}=f_{k,\pm }=0$, which indicate an equipartition of the energy in different modes. The uncertainty in the tracer Equation (8) is $\sigma_{x}=0.1$. The number of tracers is L=20, which is slightly smaller than the DoF of the resolved modes and is the typical case in practice. Appendix B includes a sensitivity analysis of the prediction skill as a function of *L*, which shows qualitatively similar results as those in the main text here.

Below, a small Rossby number ϵ=0.2 is used in the perfect model. In practice, the dynamics of the slowly-varying GB modes is often known, but building an accurate model for the fast gravity modes is a challenging task, especially for estimating the phase speeds $\omega_{k,\pm }$ [29]. To mimic such a situation, an imperfect model is used here to sample and predict the gravity modes, where the model error comes from an overestimation of the Rossby number with ϵ=0.5 in the imperfect model that leads to an inaccurate $\omega_{k,\pm }$. The available observational data has only T=10 units, which is about five decorrelation times of the Fourier modes. The prediction test is taken on an independent period with 50 units, where the true signal generated from the perfect model in this period is used as the reference value to compute the prediction skill scores in the forecast experiments.

### 4.4. Conditional Sampling

**Figure 2:**
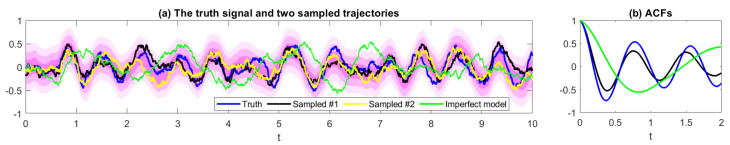
Gravity model (0,−1) of the shallow water equation. (**a**) the true signal and two sampled trajectories conditional on the observations of the tracers’ motions. The deep, moderate and light magenta shading areas show the one, two, and three standard deviations (STDs) of the uncertainty in the smoother estimate, respectively; (**b**) the ACFs of the truth, the conditional sampled trajectory and the trajectory from a free run of the imperfect model.

To expand the training data set, N=20 distinct trajectories from the conditional sampling algorithm are generated by exploiting the imperfect model, the short observations, and the formula in (4). Therefore, the effective training data for the LSTM network contains TN=200 units. Figure 2a shows the true signal and the smoother estimate of the gravity mode (0,−1), where the uncertainty in the smoother estimate facilitates the sampling of multiple trajectories. The variability in these trajectories significantly enriches the short-term time evolution patterns that are associated with the underlying flow field but are not fully reflected in the short true time series. It is also important to note that the model error in the phase speed is reduced by a large extent in the conditional sampled trajectories. In fact, (b) includes the ACF of the sampled trajectory, which demonstrates a large improvement compared with that of the imperfect model. Since the ACF characterizes the temporal dependence, the result here suggests a potential enhancement of the prediction accuracy in the LSTM forecast using the conditional sampled trajectories as training data.

### 4.5. The LSTM Setup

Recall the total DoF of the underlying flow field is 26, where the DoF of the gravity modes is 18. Since the equations of different Fourier modes are independent from each other in the perfect model, it is reasonable to build nine LSTM networks for predicting these gravity modes, each of which contains two Fourier modes that are the complex conjugate pair. Similar manipulation is used for predicting the GB modes. Since no model error is involved in the GB modes, the LSTM prediction of the GB part of the flow is almost the same as the perfect model ensemble forecast. Note that certain weak coupling between different Fourier modes may exist in the conditional sampled time series because the observations are the mixture of all the Fourier modes. Nevertheless, dividing a complex problem into several independent subproblems facilitates learning the features of the underlying dynamics. The input time series has a length of 0.25 time units while the output is the next 0.005 time units. Each of the LSTM used here contains only one hidden LSTM layer, where the number of the neurons is 100. The maximum epoch is 100. The Adam optimization algorithm is adopted, and a mean squared error loss function is used.

### 4.6. Prediction

For both the model-based ensemble forecast and the machine learning prediction here, the initial conditions of the flow fields are assumed to be perfectly known in the prediction stage. Though in practice data assimilation is required for the initializations, the idealized setup here rules out the impact from initialization and allows us to study the predictability limit of different methods.

**Figure 3:**
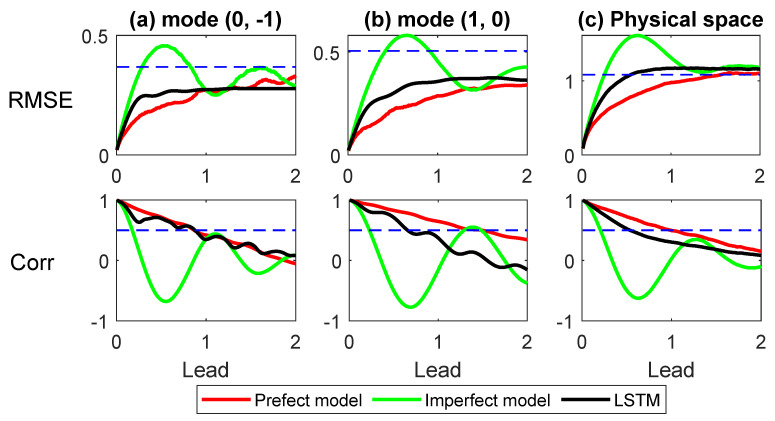
The RMSE and the Corr as a function of lead time. (**a**,**b**) the gravity modes (0,−1) and (1,0) with ζ=+. The RMSE and Corr are computed based only on the real part of the Fourier time series; (**c**) the reconstructed velocity field associated with the gravity modes in physical space, where the skill scores are the averaged values of the *u* and *v* components. In reconstructing the velocity field in physical space, a 25×25 mesh grid is used. The red, green, and black curves show the prediction skill scores using the perfect model, the imperfect model, and the LSTM network. The dashed lines in the RMSE panels indicate the one standard deviation of the true signal and those in the Corr panels show the Corr =0.5 threshold.

Figure 3 shows the root-mean-square error (RMSE) and the pattern correlation (Corr) of the predicted time series related to the truth as a function of lead time. These skill scores for the gravity modes (0,−1) and (1,0) with ζ=+ are included in (a,b). The prediction of the other gravity modes has similar behavior. (c) shows the reconstructed velocity field associated with the gravity modes in physical space. Clearly, the skill scores of the LSTM prediction are quite close to those of the perfect model, while the imperfect model has a much larger forecast bias. The improvement in the LSTM prediction is due to the significant reduction of the model error and the extended length of the training time series resulting from the conditional sampling algorithm.

**Figure 4:**
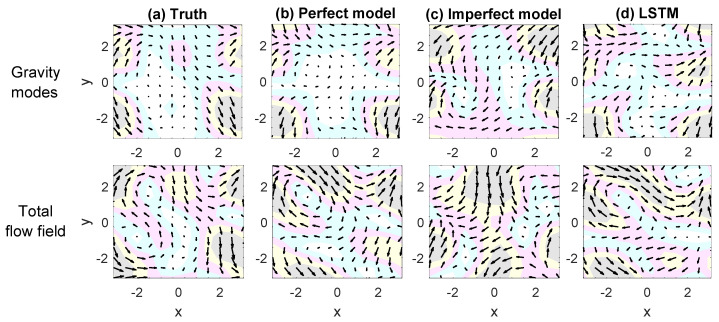
Predicting the flow fields at a lead time $t_{lead}=0.4$ in physical space. The prediction starting from t=12 in the prediction phase. (**a**–**d**) compare the truth, the perfect model forecast, the imperfect model forecast, and the LSTM forecast. The first row shows the velocity field associated with only the gravity modes while the second row shows the total velocity field. The velocity field in each panel is shown by the quivers and its amplitude is denoted by the shading area.

A case study is included in Figure 4, which compares the predicted flow fields at a lead time $t_{lead}=0.4$. The first row shows the truth and the predicted velocity field associated with only the gravity modes while the second row shows those of total velocity field that includes both the GB and gravity modes. The perfect model succeeds in predicting both the compressible gravity flows and the total velocity field. In contrast, the predicted flow pattern associated with the gravity modes from the imperfect model is completely reversed. As a result, the imperfect model fails to forecast the vortices in the total flow field in that they are overwhelmed by the large error from the forecasted gravity waves. On the other hand, despite a slight overestimation of the flow amplitude, the overall patterns are forecasted accurately by the LSTM network. In particular, both the meandering jets and the predominant vortices in the total velocity field are precisely predicted by the LSTM network.

## 5. Predicting the Monsoon Intraseasonal Oscillation (MISO)

### 5.1. The MISO Index and the Low-Order Nonlinear Stochastic Models

**Figure 5:**
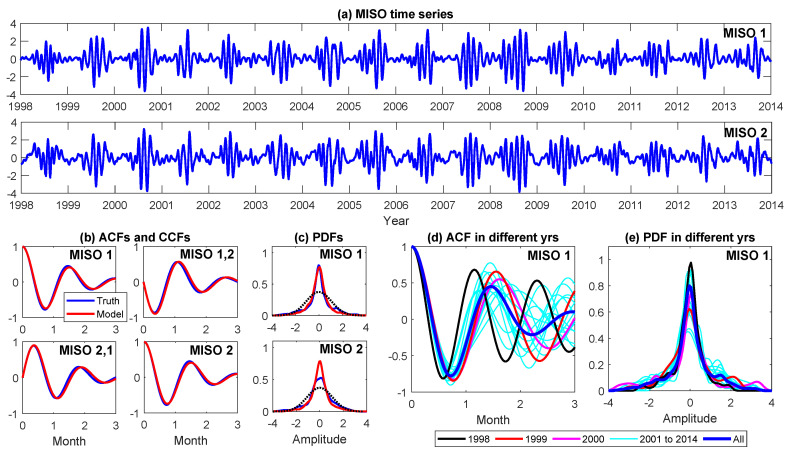
MISO index and the associated statistics. (**a**) the two components of the MISO index; (**b**) the ACFs and the cross-correlation functions (CCFs) associated with the MISO time series in (**a**) and those from the simulation of the model (9) and (10) with the parameters listed in the top row of Table 1; (**c**) similar to (**b**) but for the PDFs; (**d**) the ACF of the MISO 1 component in different years; (**e**) similar to (**d**) but for the PDF.

Monsoon Intraseasonal Oscillation (MISO) [30,31,32] is one of the prominent modes of tropical intraseasonal variability. As a slow moving planetary scale envelope of convection propagating northeastward, it strongly interacts with the boreal summer monsoon rainfall over south Asia. The MISO plays a crucial role in determining the onset and demise of the Indian summer monsoon as well as affecting the seasonal amount of rainfall over the Indian subcontinent. Therefore, both real-time monitoring and accurate extended-range forecast of MISO phases have large ecological and societal impacts. Recently, a new MISO index [33], by applying a new nonlinear data analysis technique to the daily Global Precipitation Climatology Project (GPCP) rainfall data [34] over the Asian summer monsoon region (20${}^{\circ }$ S–30${}^{\circ }$ N, 30${}^{\circ }$ E–140${}^{\circ }$ E), was developed. This new index outweighs the traditional ones that are based on the extended empirical orthogonal function (EEOF) in capturing the intermittency and nonlinear features of the MISO. The associated MISO modes also have higher memory and predictability, stronger amplitude, and higher fractional explained variance over the western Pacific, Western Ghats, and adjoining Arabian Sea regions, and more realistic representation of the regional heat sources over the Indian and Pacific Oceans compared with those extracted via EEOF analysis. (a) of Figure 5 shows this MISO index, which is a two-dimensional time series. Both components are intermittent with active phases in summer and quiescent phases in winter. The associated PDFs, as are shown in the blue curves, are highly non-Gaussian.

To develop a model that describes such a MISO index, it is natural to start with a two-dimensional linear stochastic oscillator. However, the linear oscillator model itself is not sufficient to characterize all the observed features of the observed MISO index. In particular, it fails to capture the variability in the amplitude and the oscillation frequency. In fact, as is shown in (d–e) in Figure 5, the dynamical and statistical behavior of the MISO index in different years is quite distinct with each other. Therefore, a simple but effective way to take into account these characteristics is to add two extra processes, representing the randomness in damping and phase [35]. The model reads

(9) $$\begin{array}{cc} \;du_{1} & =\left(-d_{u}u_{1}+\gamma \left(v+v_{f}\left(t\right)\right)u_{1}-\left(a+\omega \right)u_{2}\right)\;dt+\sigma_{u}\;dW_{u_{1}},\\ \;du_{2} & =\left(-d_{u}u_{2}+\gamma \left(v+v_{f}\left(t\right)\right)u_{2}+\left(a+\omega \right)u_{1}\right)\;dt+\sigma_{u}\;dW_{u_{2}},\\ \;dv & =-d_{v}v\;dt+\sigma_{v}\;dW_{v},\\ \;d\omega & =-d_{\omega }\omega \;dt+\sigma_{\omega }\;dW_{\omega }, \end{array}$$

where the time-periodic function

(10) $$v_{f}\left(t\right)=f_{0}+f_{1}\sin\left(\omega_{f}t+\phi \right)$$

provides a crude description of the seasonal cycle. In this model, $u_{1}$ and $u_{2}$ stand for the two components of the MISO index while *v* and ω are the stochastic damping and the stochastic phase, respectively. The white noise sources $W_{u_{1}}$, $W_{u_{2}}$, $W_{v}$ and $W_{\omega }$ are independent from each other. Note that the nonlinear model developed here is slightly different from the physics-constraint model in [35]. However, these two models have almost the same skill in predicting the MISO index. The main reason to adopt the form in (9) and (10) is that the model structure of this coupled nonlinear model facilitates the application of the conditional sampling algorithm.

**Figure A3:**
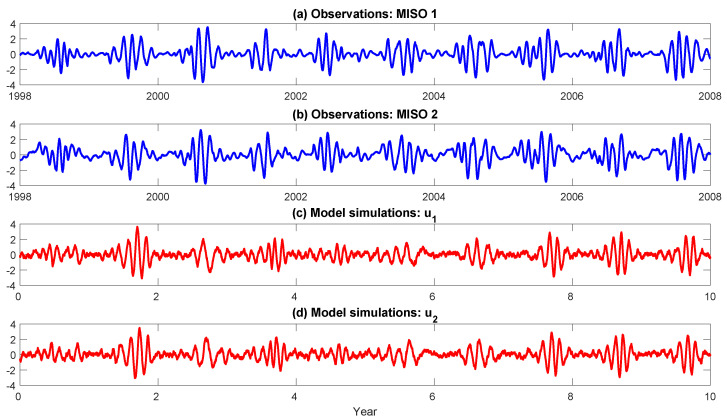
(**a**,**b**) the two components of the observed MISO index (the same as those in Figure 5); (**c**,**d**) a simulation from the nearly perfect model (9) and (10), where the parameters are listed in the top row of Table 1.

To illustrate the skill of the coupled model (9) and (10) in capturing the nonlinear and non-Gaussian features of the MISO index, it is shown in (b,c) of Figure 5 that the model can perfectly recover the ACFs, the cross-correlation functions (CCFs), and the non-Gaussian PDFs of the entire MISO index. The model trajectories also highly resemble the true MISO index (see Figure A3 in Appendix C). The associated model parameters are listed in the top row of Table 1. Due to the high skill in describing the MISO index, the model (9) and (10) with these parameters is named as the “nearly perfect model”.

It is important to note that the nearly perfect model is obtained by exploiting *all* the observational information (1998–2013) for model calibration. Therefore, despite the fact that such a model succeeds in describing nature, it cannot be used for predicting the MISO index during the same period because of the overfitting issue. In the following, only the MISO index in year 1998 is used for model calibration. This mimics most applications in nature where the data for model calibration is very limited. On the other hand, the time series from year 2001 to year 2013 is adopted for testing the prediction skill, which provides a sufficient long validation period to reach a robust conclusion.

Due to the fact that the MISO index has distinct year-to-year behavior ((d,e) in Figure 5), the model parameters estimated by using only the time series in year 1998 are different from the nearly perfect model. These estimated model parameters are shown in the bottom row of Table 1 and the resulting model is named as the “imperfect model” in predicting the MISO time series from year 2001 to year 2013. Below, either a one-year or a three-year time series is combined with this imperfect model to enrich the training data set for the machine learning forecasts by applying the conditional sampling technique. Note that, although the imperfect model can be in principle re-calibrated when the new data comes sequentially in time, the parameters in the imperfect model are unchanged in the tests here for two reasons. First, the online parameter estimation for complex nonlinear systems in real applications can be extremely expensive and thus it is seldom adopted in practice. Second, there often exists an intrinsic model error in real applications. The imperfect parameters here mimic such an intrinsic barrier since otherwise the model is nearly perfect.

### 5.2. Conditional Sampling

Below, the short observational data in (1) year 1998, (2) year 1999, (3) year 2000, or (4) a three-year period 1998–2000 are utilized together with the imperfect model to enrich the training data set of the machine learning forecast based on the conditional sampling technique. The conditional sampling algorithm here involves two steps:

Step 1. Conditioned on the two components $X={\left(u_{1},u_{2}\right)}^{T}$ of the observed MISO index, sample *N* trajectories of $Y={\left(v,\omega \right)}^{T}$.
Step 2. Conditioned on each of the sampled trajectories, denoted now by $X={\left(v,\omega \right)}^{T}$, from Step 1, sample a trajectory of $Y={\left(u_{1},u_{2}\right)}^{T}$.

In both the steps, X denotes the conditional variables while Y stands for the sampled variables. The structure of the nonlinear model (9) and (10) allows the setups in both the steps belong to the nonlinear modeling family (1) such that the closed analytic Formulae (4) can be applied in sampling both (v,ω) and $\left(u_{1},u_{2}\right)$. Note that, despite $\left(u_{1},u_{2}\right)$ being not appeared in the equations of (v,ω), the conditional sampling in step 2 is necessary since uncertainty exists in the processes of (v,ω). Simply plugging the sampled trajectories of (v,ω) into the equations of $\left(u_{1},u_{2}\right)$ leads to biased results.

### 5.3. The Setup of the LSTM and the Model Ensemble Forecast

In all the tests here, a 30-year sampled time series of $\left(u_{1},u_{2}\right)$ is used as the machine learning training data. This implies N=30 when a one-year observational data are used in the conditional sampling and N=10 when the three-year data from year 1998 to year 2000 is used. The hidden variables (v,ω) are only used to generate the sampled trajectories of $\left(u_{1},u_{2}\right)$, but they are not directly utilized in the machine learning training and forecasting steps. The input data $\left(u_{1},u_{2}\right)$ in the LSTM has a length of 30 days and the output is the next one day. The number of the neurons in the LSTM layer is 200, and the maximum epoch is 100.

The model ensemble forecast is adopted as a comparison with the machine learning prediction. The model ensemble forecast exploits 50 ensemble members, which have been validated to provide an unbiased ensemble mean prediction time series. Since there is no observational data for the two hidden variables *v* and ω, the exact data assimilation scheme in (2) is utilized for their initializations.

### 5.4. Prediction

**Figure 6:**
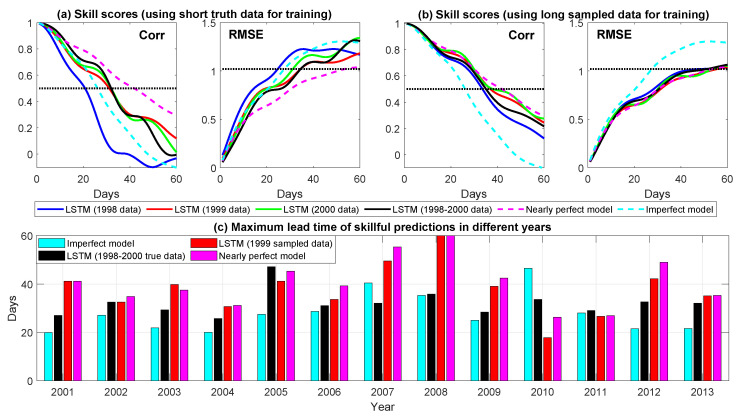
The MISO prediction. (**a**) the RMSE and Corr as a function of lead time (days) in predicting the MISO time series from 2001 to 2013, where the short observational data are used for training the LSTM. Here, the solid blue, red, and green curves are the LSTM prediction based on a one-year training data using the observed time series in year 1998, 1999, and 2000, respectively. The solid black curve is the LSTM prediction based on a three-year training data using the observed time series from 1998 to 2000. The dashed pink and cyan curves show the model predictions using the nearly perfect model and the imperfect model. The black dotted lines show the threshold Corr=0.5 and one standard deviation of the true time series. (**b**) is similar to (**a**), but the training data of the LSTM are obtained by applying the conditional sampling algorithm to the observed time series in either one of the three years of 1998 (blue), 1999 (red) and 2000 (green), or all three years (black). The total length of the sampled data are 30 years; (**c**) the maximum lead time of the skillful prediction in different years. Here, the skillful prediction is defined as the Corr>0.5 and RMSE<1STDSTD (standard deviation) of the true signal.

The MISO index prediction using different methods is shown in Figure 6. The solid curves in both (a) and (b) illustrate the overall prediction skill in the 2001–2013 period using the LSTM. The difference between these two panels is the following: In (a), the training data are the short observational data with either one year (blue, red, and green curves) or three years (black curves). On the other hand, in (b), the 30-year sampled time series is used for the LSTM training. As a comparison, the model ensemble forecast results are also included in these panels, where the dashed pink curve shows the prediction using the nearly perfect model while the dashed cyan curve illustrates this using the imperfect model.

**Figure A4:**
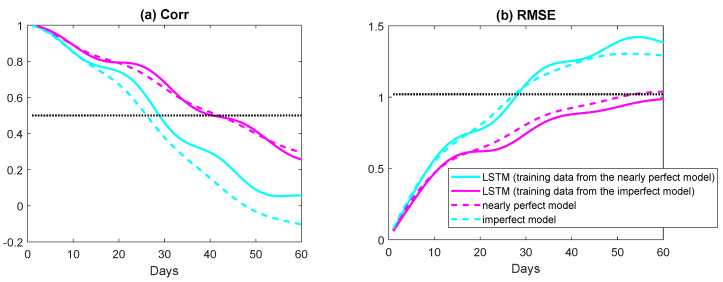
Skill scores of predicting the MISO index. The two panels here are similar to those in Figure 6, but the solid curves here show the training data of the LSTM is from the model simulation with a 30-year length. The dashed curves show the model ensemble forecasts.

The main conclusions are as follows: first, either a one-year or a three-year time series is not sufficient for training the LSTM. Among all these short training data, using the time series in year 1998 brings about the worst prediction skill. This is because the dynamical and statistical features of the MISO index in year 1998 are more distinguished from those in the entire observational period, compared with year 1999 and year 2000 (see (d,e) in Figure 5). The consequence is that the LSTM prediction using only the time series of year 1998 as the training data performs even significantly worse than the ensemble forecast using the imperfect model. Second, the LSTM prediction using the long sampled trajectories based on the information of the extremely short observations in year 1999 or year 2000 has almost the same skill as the perfect model forecast! In fact, the medium-range forecast (20 to 30 days) using the LSTM even slightly outweighs that using the perfect model. Notably, the LSTM prediction using the long sampling data based on the time series of year 1998 is also greatly improved and outweighs the ensemble forecast using the imperfect model. This seems to be counter-intuitive. Nevertheless, since the model is stochastic, it is able to generate patterns with different oscillation frequencies in the conditional sampled trajectories. The LSTM forecast then exploits the input information to find the most similar patterns in the training data set. In other words, the LSTM gives different weights to all the possible events in the training data set, which can be regarded as different ensemble members. This is, however, not the case in the model based ensemble mean forecast, where the same weight is assigned to all the ensemble members. Thus, despite making use of the same observational information, the LSTM performs better than the model ensemble mean forecast. In Figure A4 of Appendix C, the model generated training data are used for the LSTM prediction, which also indicates such a mechanism.

**Figure 7:**
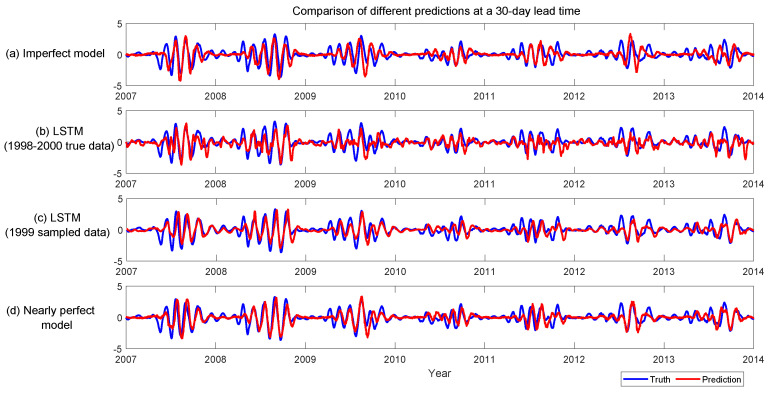
Comparison of the predicted MISO index using different methods. (**a**) prediction using the imperfect model; (**b**) prediction using the LSTM, where the training data are the true observational data from 1998 to 2000; (**c**) prediction using the LSTM, where the training data are obtained by applying the conditional sampling algorithm to the observed MISO time series of year 1999 with in total 30 years sampled data; (**d**) prediction using the nearly perfect model.

(e) of Figure 6 shows the maximum lead time of skillful predictions in different years. Here, the skillful prediction is defined as the Corr>0.5 and RMSE<1STD (standard deviation) of the true signal. Although the useful predictions vary from year to year, the LSTM prediction using the conditional sampled trajectories based on the one-year observed time series in 1999 is comparable to the nearly perfect model forecast. The associated maximum lead time is longer than that using the three-year short training data and the imperfect model forecast in most of the years. The only exception is year 2010. In fact, the MISO pattern of year 2010 is similar to that of year 1998 with a weak amplitude. Note that the winter of both year 1998 and year 2010 corresponds to a strong positive phase of the Atlantic Zonal Mode (AZM) [36,37]. The results here confirm the negative correlation between the MISO and the AZM as well as the relatively short predictability of the MISO at the positive phase of the AZM. Next, 2002 and year 2004 are recorded as drought years. The MISO index in these two years is more irregular than the other years, which explains its lower overall prediction skill than most of the other years. On the other hand, 2008 has an overall strong and regular MISO activity during the whole monsoon season that results in a long predictability. Finally, Figure 7 shows the predicted time series at the lead time of 30 days, which clearly indicates the LSTM with the conditional sampled training data outperforms the imperfect model ensemble forecast in most of the years.

## 6. Conclusions

An efficient and optimal algorithm for sampling nonlinear time series conditioned on the observations is developed in this article. It exploits only short and partial observations to greatly reduce the model error and expand the training data set for the machine learning forecast algorithms. The sampling algorithm succeeds in creating a large training data of multiscale compressible shallow water flows from highly nonlinear and indirect observations. The resulting machine learning prediction significantly outweighs the imperfect model forecast. The sampling algorithm also facilitates the machine learning forecast of the highly non-Gaussian MISO time series using extremely short observations.

Note that the MISO is an intraseasonal variability and therefore the total available data of MISO in the satellite era is actually not that restricted. The reason to adopt only a small portion of the observed MISO data for training is to mimic the real situations of predicting many other nature phenomena. Meanwhile, the remaining relatively long MISO trajectories let us study the improvement of the MISO forecast using the conditional sampling technique. On the other hand, the El Niño-Southern Oscillation (ENSO) is an interannual variability, which has significant impact on the entire earth system. The ENSO has various spatiotemporal patterns, but the observational data of the ENSO is very scarce. In addition, the current operational models have large model error in describing and predicting the ENSO. Thus, an interesting and useful task is to utilize the conditional sampling algorithm for improving the ENSO prediction, which is left as a future work. Another future direction is to generalize the conditional sampling algorithm to general nonlinear and non-Gaussian systems using particle methods.

## Figures and Tables

**Table 1:** Two sets of the parameters for the model (9) and (10).

	$d_{u}$	$d_{v}$	$d_{\omega }$	γ	*a*	$\sigma_{u}$	$\sigma_{v}$	$\sigma_{\omega }$	$f_{0}$	$f_{1}$	$\omega_{f}$	ϕ
I. nearly perfect model	0.9	0.6	0.5	0.2	4.1	0.5	0.5	0.7	1	4.7	2π/12	−2
II. imperfect model	0.9	0.6	0.5	0.2	5.2	0.5	0.5	0.7	1	4.7	2π/12	−2

**Figure A1:**
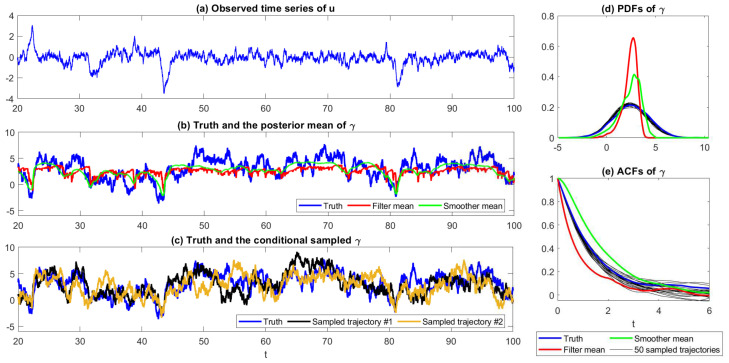
(**a**) the true signal of *u*; (**b**) the true signal of γ (blue), the filter posterior mean time series (red) and the smoother posterior mean time series (green); (**c**) the true signal of γ (blue) and two sampled trajectories (black and brown); (**d**,**e**) comparison of the PDFs and ACFs of γ. The blue curve is the PDF associated with the true signal. The red and green curves are the PDFs associated with the filter and smoother posterior mean time series, respectively. The black curves are the PDFs associated with 50 trajectories from the conditional sampling. These statistics are computed based on time series with 1000 time units, which are sufficiently long to rule out almost all the undersampling errors.

**Figure A2:**
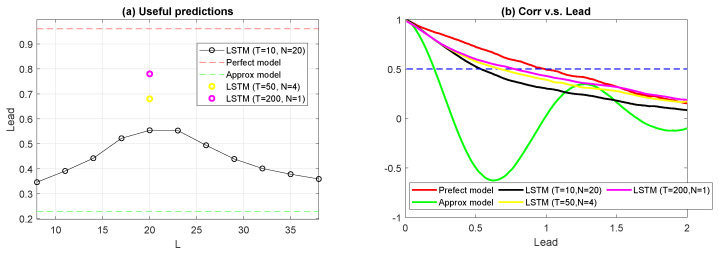
Sensitivity analysis. (**a**) the black curve shows the maximum lead time of the useful prediction using the LSTM network as a function of the number of tracers *L* with T=10 and N=20. Here, the useful prediction is defined as the lead time, within which the RMSE is less than the one standard deviation of the true signal and the Corr is above the threshold Corr =0.5. The yellow and pink circles show the lead time of the useful prediction using the LSTM network with T=50,N=4 and T=200,L=1, respectively. The red and green dashed lines show the useful predictions using the perfect and the imperfect models, which do not depend on *L* when the initial values are perfectly known; (**b**) the Corr as a function of the lead time using different methods.

## Appendix A. Conditional Sampling

### Appendix A.1. An Alternative Conditional Sampling Formula That Requires Only the Filter Estimates

Recall the conditional sampling formula in the main text (Theorem 3), which is mathematically concise as is discussed in the main text. However, it requires the information of both the filter and smoother. An alternative form that makes use of only the filter estimate is given as follows, which is more computationally convenient.

**Corollary** **A1** (An Alternative Form of the Conditional Sampling Formula)**.**

(A1) $$\overset{\leftarrow }{\;dY}=\left(-a_{0}-a_{1}Y\right)\;dt+\left(b\circ b\right)R_{f}^{-1}\left(\mu_{f}-Y\right)\;dt+{\left(b\circ b\right)}^{1/2}\;dW_{Y},$$

Below is a simple proof of the equivalence of Theorem 3 and Corollary A1.

**Proof.** Recall the smoother posterior mean equation

(A2) $$\overset{\leftarrow }{\;d\mu_{s}}=\left(-a_{0}-a_{1}\mu_{s}+\left(b\circ b\right)R_{f}^{-1}\left(\mu_{f}-\mu_{s}\right)\right)\;dt.$$

Taking the summation of (4) and (A2) cancels $\overset{\leftarrow }{\;d\mu_{s}}$ and $\mu_{s}$. The resulting equation yields

(A3) $$\begin{array}{cc} \overset{\leftarrow }{\;dY}-\overset{\leftarrow }{\;d\mu_{s}} & =-a_{1}\left(Y-\mu_{s}\right)\;dt+\left(b\circ b\right)R_{f}^{-1}\left(\mu_{s}-Y\right)\;dt+{\left(b\circ b\right)}^{1/2}\;dW_{Y}\\ \; & =-\left(a_{1}+\left(b\circ b\right)R_{f}^{-1}\right)\left(Y-\mu_{s}\right)\;dt+{\left(b\circ b\right)}^{1/2}\;dW_{Y}, \end{array}$$

which is (A1).  □

### Appendix A.2. Comparison of the Posterior Mean Time Series and the Trajectories from Conditional Sampling in Reproducing the Dynamical and Statistical Characteristics of Nature

The goal of this subsection is to show that, even in the perfect model setup, the posterior mean time series fails to reproduce the basic dynamical and statistical features of nature. This implies that the machine learning prediction can be biased if the training is based on the posterior mean time series.

Consider a simple example

(A4a) $$\begin{array}{cc} \;du & =\left(-\gamma u+f_{u}\right)\;dt+\sigma_{u}\;dW_{u} \end{array}$$

(A4b) $$\begin{array}{cc} \;d\gamma & =\left(-d_{\gamma }\gamma +u^{2}+f_{\gamma }\right)\;dt+\sigma_{\gamma }\;dW_{\gamma } \end{array}$$

with the following parameters

(A5) $$\sigma_{u}=1,\;{\;d}_{\gamma }=0.5,\;f_{\gamma }=0.8,\;\sigma_{\gamma }=2.$$

This is a simple nonlinear dyad model with energy conserving nonlinearity (known also as physics constraints) [38,39]. The variable *u* is observed while the hidden variable *v* plays the role of the stochastic damping that triggers intermittent instability and extreme events in *u*. See the burst events in (a) of Figure A1.

Now conditioned on one observed trajectory of *u*, the filter and smoother posterior mean time series of *v* are shown in (b) of Figure A1. At the phases when extreme events occur in *u*, the smoother posterior mean succeeds in recovering the true signal, but the filter posterior mean fails to predict the timing of the event onset and the duration. However, when the observed signal of *u* is at its quiescent phases, the small signal-to-noise ratio leads to a large error in the posterior mean time series with a large uncertainty. The dynamical behavior of the posterior mean time series is obviously very different from the truth. It is thus expected that the machine learning prediction can be biased by using these time series as the training data. In fact, as is shown in (d)–(e), both the PDFs and ACFs associated with the posterior mean time series (regardless of filter or smoother) are biased from the truth. The large error in the PDFs from (d) is as expected since the posterior mean time series of γ in (b) essentially follows the model’s equilibrium state at the quiescent phases of *u* and fails to capture the variability of the truth. Its consequence to the machine learning prediction is that the events of γ do not appear in the training period (the posterior mean time series) will never be forecasted and therefore the forecast contains a large error. On the other hand, the ACFs associated with the posterior mean time series are also biased from the truth according to (e). This can be seen intuitively from the time series in (b). Since the ACF represents the averaged path-wise memory of the system, the error in the ACFs affects the short-term prediction. In contrast, the sampled trajectories from the conditional sampling perfectly recover both the PDFs and the ACFs. This indicates that the sampled trajectories are able to fully recover both the dynamical and statistical features of nature. Therefore, training the machine learning algorithms using these sampled trajectories are expected to provide skillful prediction results.

(c) shows two sampled trajectories. It is clear that these sampled trajectories have similar dynamical and statistical features as the truth. In addition, since they are calculated by conditioning on the observations, they are different from a free run of the model. This is an extremely important feature when model error comes, at which time the observations can mitigate the model error and the resulting sampled trajectories will be more accurate than the model free run. On the other hand, the sampled trajectories may have a potential to facilitate the prediction of rare and extreme events. In fact, despite a large variability in the sampled trajectories of γ at the quiescent phases of *u*, the uncertainty in the sampled trajectories corresponding to the extreme events of *u* is small. This means the phases corresponding to the extreme events are guaranteed to be sampled in these trajectories. Meanwhile, the sampled trajectories themselves have an extra chance to reach the phases corresponding to extreme events—for example the event at time instants t=98 on the brown curves.

## Appendix B. Details of Predicting Multiscale Compressible Shallow Water Flows Using Lagrangian Observations

### Appendix B.1. Details of the Shallow Water Equation

Recall the two-dimensional linear rotating shallow water Equation (5) in the main text, where the solution is given by

(A6) $$\left(\begin{array}{c} v(x,t)\\ \eta (x,t) \end{array}\right)=\sum_{k\in Z^{2},\zeta \in \left\{B,\pm \right\}}{\hat{u}}_{k,\zeta }\left(t\right)e^{ik\cdot x}r_{k,\zeta }.$$

In (A6), the modes with ζ=B are the geostrophically balanced (GB) modes, where the GB relation $u^{⊥}=-\nabla \eta$ always holds. The GB flows are incompressible, where the incompressibility ∇·u=0 is embodied in the eigenvector $r_{k,\zeta }$, and the associated phase speed is $\omega_{k,B}=0$. The modes with ζ=± represent the gravity modes (also known as the Poincaré waves). The associated phase speed is $\omega_{k,\pm }=\pm \epsilon^{-1}\sqrt{{\left|k\right|}^{2}+1}$, and they are compressible. The normalized eigenvector of the GB modes $r_{k,B}$ is given by

(A7) $$r_{k,B}=\frac{1}{\sqrt{{\left|k\right|}^{2}+1}}\left(\begin{array}{c} -ik_{2}\\ ik_{1}\\ 1 \end{array}\right).$$

The normalized eigenvectors $r_{k,\pm }$ of the gravity modes are given by

(A8) $$r_{k,\pm }=\frac{1}{\left|k\right|\sqrt{\left(\delta +\delta^{2}\right){\left|k\right|}^{2}+2}}\left(\begin{array}{c} ik_{2}\pm k_{1}\sqrt{{\delta |k|}^{2}+1}\\ -ik_{1}\pm k_{2}\sqrt{{\delta |k|}^{2}+1}\\ {\delta |k|}^{2} \end{array}\right)$$

For the special case, k=0, the Poincaré waves have no gravity component and coincide with the inertial waves. The resulting eigenvalues become $r_{0,\pm }=\pm \epsilon^{-1}$ with the eigenvectors being

(A9) $$r_{0,\pm }=\frac{1}{\sqrt{2}}\left(\begin{array}{c} \pm i\\ 1\\ 0 \end{array}\right).$$

Note from the eigenvector of the GB modes (A7) that the first two components of the (0,0)-th mode $r_{0,B}$ are zero. This mode often represents the given background flow. Since it has no contribution to the observational process, it is removed here. Recall that the modes with Fourier wavenumbers $-1\leq k_{1},k_{2}\leq 1$ are used in the main text. Therefore, the total degree of freedom (DoF) of the underlying flow model is DoF = 26, which includes 8 GB modes and 2×9 gravity modes.

### Appendix B.2. Sensitivity Analysis

Sensitivity analysis is carried out in this subsection to study the dependence of the prediction skill on several key parameters.

One of the important parameters for the sensitivity study is the number of the Lagrangian tracers *L*. To begin with, it is expected that the LSTM network prediction can become less accurate in the two extreme situations with either a very large or a very small *L*. In fact, when L=1, the information provided by the observation is very limited. As a result, the LSTM prediction is only slightly better than the forecast based on the imperfect model. On the other hand, when *L* becomes infinity, the posterior estimate converges to the true short signal with no uncertainty. However, a short training period is not sufficient for training the LSTM network, which therefore leads to the deterioration of the prediction skill. In such a situation, the LSTM network prediction is even worse than the imperfect model forecast. Figure A2a shows the maximum lead time of the useful prediction of the LSTM network as a function of *L*. Here, the useful prediction is defined as the lead time, within which the RMSE is less than the one standard deviation of the true signal and the Corr is above the threshold Corr =0.5. It is seen that, within a relatively wide range L∈[17,26], the LSTM network prediction is significantly more skillful than the imperfect model forecast. The LSTM network prediction remains more skillful than the imperfect model prediction by a large amount even with a further increasing or decreasing of *L*. Note that, despite the useful prediction based on the LSTM network having an obvious gap compared with that from the perfect model prediction, the overall difference in the prediction skill between these two methods is not as significant as that between the LSTM network prediction with the imperfect model forecast. In fact, as shown in (b), the skill score curves associated with both the LSTM network and the perfect model forecast flatten after a short time, which lead to the large gap in (a), but the two curves are not too far from each other.

Another key parameter for the sensitivity analysis is the length of the observational period *T*. Recall that the length of the observation is T=10 and N=20 repeated sampled trajectories are used to form the training data set of the LSTM network in the main text. Two extra experiments are carried out here with T=50 and N=4 (yellow circle/curve) and T=200 and N=1 (pink circle/curve). It turns out that, within $t_{lead}=0.3$, the prediction skill with T=10 is nearly the same as those with T=50 and T=200. As the lead time increases, the Corr with T=50 and T=200 becomes slightly higher than the short observational case with T=10, as is expected. Nevertheless, comparing with the enhanced prediction skill from using the imperfect model (green curve) to the LSTM forecast with T=10, the above improvement is relatively marginal. Therefore, these tests indicate that a short observation with T=10 is sufficient to allow a skillful prediction using the LSTM network since the multiple sampled trajectories include a large variability of the underlying systems.

## Appendix C. Additional Results for Predicting the MISO Index

(c,d) of Figure A3 show a simulation from the nearly perfect model (9) and (10). The path-wise behavior of the model resembles the observed MISO index, which is shown in (a,b).

Figure A4 shows the skill scores of the MISO prediction. the training data of the LSTM in this figure is provided by the model simulation. Here, the model simulation means simply running the model (9) and (10) forward for 30 years. There is no observational data incorporated here and there is no conditional sampling either. The following conclusions can be drawn from this figure. If the nearly perfect model is used to provide a massive training data, then the skill scores of the LSTM prediction are comparable to those of the model ensemble forecast using the nearly perfect model. On the other hand, if the imperfect model is used to generate the training data, then the pattern correlation of the LSTM prediction outweighs the imperfect model ensemble forecast. The underlying reason is the following. Since the model is a stochastic model, it is able to generate patterns with different oscillation frequencies. The LSTM forecast then exploits the input information to find the most similar patterns in the training data set. In other words, the LSTM gives different weights to all the possible events in the training data set, which can be regarded as different ensemble members. This is, however, not the case in the ensemble mean based model forecast, where the same weight is assigned to different ensemble members.

## Appendix D. The Derivation of the Conditional Sampling Formula

For the convenience of discussion, the statement below starts with a discrete approximation of the original nonlinear continuous system in time (1) by adopting a Euler–Maruyama scheme,

(A10) $$\begin{array}{cc} {\overset{˜}{X}}^{j+1} & ={\overset{˜}{X}}^{j}+\left({\overset{˜}{A}}_{0}^{j}+{\overset{˜}{A}}_{1}^{j}{\overset{˜}{Y}}^{j}\right)\Delta t+{\overset{˜}{B}}_{1}^{j}\Delta {\overset{˜}{W}}_{1}^{j}+{\overset{˜}{B}}_{2}^{j}\Delta {\overset{˜}{W}}_{2}^{j},\\ {\overset{˜}{Y}}^{j+1} & ={\overset{˜}{Y}}^{j}+\left({\overset{˜}{a}}_{0}^{j}+{\overset{˜}{a}}_{1}^{j}{\overset{˜}{Y}}^{j}\right)\Delta t+{\overset{˜}{b}}_{1}^{j}\Delta {\overset{˜}{W}}_{1}^{j}+{\overset{˜}{b}}_{2}^{j}\Delta {\overset{˜}{W}}_{2}^{j}, \end{array}$$

Thus, the values of X and Y are taken at discrete points in time $\left\{{\overset{˜}{X}}^{0},\ldots ,{\overset{˜}{X}}^{j},\ldots ,{\overset{˜}{X}}^{J}\right\}$ and $\left\{{\overset{˜}{Y}}^{0},\ldots ,{\overset{˜}{Y}}^{j},\ldots ,{\overset{˜}{Y}}^{J}\right\}$, where ${\overset{˜}{X}}^{j}:=X\left(t_{j}\right)$ and ${\overset{˜}{Y}}^{j}=Y\left(t_{j}\right)$. Here, the variable with tilde and superscript *j*, namely ${\overset{˜}{\cdot }}^{j}$, denotes the discrete approximation of its continuous form at time $t_{j}$, where the entire time interval [0,T] is divided into *J* equipartition subintervals with $0=t_{0},t_{1},t_{2},\ldots ,t_{J}=T$. Denote $\Delta t=t_{j+1}-t_{j}$ and therefore JΔt=T. In the analysis of the system with the discrete approximation, Δt is assumed to be a small value. At the end, the limit Δt→0 will be taken for the discrete approximation to retrieve the original continuous dynamics.

Different from the point-wise state estimation (i.e., filtering or smoothing), which is only conditioned on the observations, calculating the current value ${\overset{˜}{Y}}^{j}$ on the conditional sampled trajectory requires the given conditions on both the observations ${\overset{˜}{X}}^{s},s\leq j$ and the previous sampled point ${\overset{˜}{Y}}^{j+1}$. Therefore, the following Lemma is useful for deriving the conditional sampling formula.

**Lemma** **A1.**
*The conditional distribution*

(A11) $$p\left({\overset{˜}{Y}}^{j}|{\overset{˜}{Y}}^{j+1},{\overset{˜}{X}}^{s},s\leq j\right)∼N\left({\overset{˜}{m}}^{j},{\overset{˜}{P}}^{j}\right)$$

*is Gaussian, where the conditional mean ${\overset{˜}{m}}^{j}$ and conditional covariance ${\overset{˜}{P}}^{j}$ satisfies the following equations:*

(A12a) $$\begin{array}{cc} {\overset{˜}{m}}^{j} & ={\overset{˜}{\mu }}^{j}+{\overset{˜}{C}}^{j}\left({\overset{˜}{Y}}^{j+1}-{\overset{˜}{a}}_{0}^{j}\Delta t-\left(I+{\overset{˜}{a}}_{1}^{j}\Delta t\right){\overset{˜}{\mu }}^{j}\right), \end{array}$$

(A12b) $$\begin{array}{cc} {\overset{˜}{P}}^{j} & ={\overset{˜}{R}}^{j}-{\overset{˜}{C}}^{j}\left({\overset{˜}{b}}^{j}\circ {\overset{˜}{b}}^{j}\Delta t+\left(I+{\overset{˜}{a}}_{1}^{j}\Delta t\right){\overset{˜}{R}}^{j}{\left(I+{\overset{˜}{a}}_{1}^{j}\Delta t\right)}^{*}\right){\left(C^{j}\right)}^{*}, \end{array}$$

*and the auxiliary matrix C is given by*

(A13) $${\overset{˜}{C}}^{j}={\overset{˜}{R}}^{j}{\left(I+{\overset{˜}{a}}_{1}^{j}\Delta t\right)}^{*}{\left({\overset{˜}{b}}^{j}\circ {\overset{˜}{b}}^{j}\Delta t+\left(I+{\overset{˜}{a}}_{1}^{j}\Delta t\right){\overset{˜}{R}}^{j}{\left(I+{\overset{˜}{a}}_{1}^{j}\right)}^{*}\right)}^{-1}.$$

**Proof.** The conditional distribution $p({\overset{˜}{Y}}^{j}|{\overset{˜}{Y}}^{j+1},{\overset{˜}{X}}^{s},s\leq j)$ can be solved by making use of the joint distribution $p({\overset{˜}{Y}}^{j},{\overset{˜}{Y}}^{j+1}|{\overset{˜}{X}}^{s},s\leq j)$. In light of the second equation in (A10), the marginal distribution $p({\overset{˜}{Y}}^{j+1}|{\overset{˜}{X}}^{s},s\leq j)$ is given by

(A14) $$p\left({\overset{˜}{Y}}^{j+1}|{\overset{˜}{X}}^{s},s\leq j\right)∼N\left({\overset{˜}{a}}_{0}^{j}\Delta t+\left(I+{\overset{˜}{a}}_{1}^{j}\Delta t\right)\overset{˜}{\mu },\;{\overset{˜}{b}}^{j}\circ {\overset{˜}{b}}^{j}\Delta t+\left(I+{\overset{˜}{a}}_{1}^{j}\Delta t\right){\overset{˜}{R}}^{j}{\left(I+{\overset{˜}{a}}_{1}^{j}\Delta t\right)}^{*}\right).$$

On the other hand, the marginal distribution $p({\overset{˜}{Y}}^{j}|{\overset{˜}{X}}^{s},s\leq j)$ is simply given by the filtering formula,

(A15) $$p\left({\overset{˜}{Y}}^{j}|{\overset{˜}{X}}^{s},s\leq j\right)∼N\left({\overset{˜}{\mu }}^{j},{\overset{˜}{R}}^{j}\right).$$

The cross covariance term conditioned on the observations is given by

(A16) $$\langle {\overset{˜}{Y}}^{\prime ,j+1}{\left({\overset{˜}{Y}}^{\prime ,j}\right)}^{*}\rangle |{\overset{˜}{X}}^{s},s\leq j=\left(I+{\overset{˜}{a}}_{1}^{j}\Delta t\right){\overset{˜}{R}}^{j},$$

where ${\overset{˜}{Y}}^{\prime ,j+1}$ and ${\overset{˜}{Y}}^{\prime ,j}$ are ${\overset{˜}{Y}}^{j+1}$ and ${\overset{˜}{Y}}^{j}$ by removing their mean values. Therefore, collecting (A14)–(A16) leads to

(A17) $$\begin{array}{cc} \; & p({\overset{˜}{Y}}^{j},{\overset{˜}{Y}}^{j+1}|{\overset{˜}{X}}^{s},s\leq j)\\ \; & ∼N\left(\left(\begin{array}{c} {\overset{˜}{\mu }}^{j}\\ {\overset{˜}{a}}_{0}^{j}\Delta t+\left(I+{\overset{˜}{a}}_{1}^{j}\Delta t\right){\overset{˜}{\mu }}^{j} \end{array}\right),\left(\begin{array}{cc} {\overset{˜}{R}}^{j} & {\overset{˜}{R}}^{j}{\left(1+{\overset{˜}{a}}_{1}^{j}\Delta t\right)}^{*}\\ \left(I+{\overset{˜}{a}}_{1}^{j}\Delta t\right){\overset{˜}{R}}^{j} & {\overset{˜}{b}}^{j}\circ {\overset{˜}{b}}^{j}\Delta t+\left(I+{\overset{˜}{a}}_{1}^{j}\Delta t\right){\overset{˜}{R}}^{j}{\left(I+{\overset{˜}{a}}_{1}^{j}\Delta t\right)}^{*} \end{array}\right)\right). \end{array}$$

In light of the rule of the multivariate Gaussian distribution, the result in (A17) yields the conditional distribution,

(A18) $$p\left({\overset{˜}{Y}}^{j}|{\overset{˜}{Y}}^{j+1},{\overset{˜}{X}}^{s},s\leq J\right)=p\left({\overset{˜}{Y}}^{j}|{\overset{˜}{Y}}^{j+1},{\overset{˜}{X}}^{s},s\leq j\right)=N\left({\overset{˜}{m}}^{j},{\overset{˜}{P}}^{j}\right),$$

where

(A19a) $$\begin{array}{cc} {\overset{˜}{m}}^{j} & ={\overset{˜}{\mu }}^{j}+{\overset{˜}{C}}^{j}\left({\overset{˜}{Y}}^{j+1}-{\overset{˜}{a}}_{0}^{j}\Delta t-\left(I+{\overset{˜}{a}}_{1}^{j}\Delta t\right){\overset{˜}{\mu }}^{j}\right), \end{array}$$

(A19b) $$\begin{array}{cc} {\overset{˜}{P}}^{j} & ={\overset{˜}{R}}^{j}-{\overset{˜}{C}}^{j}\left({\overset{˜}{b}}^{j}\circ {\overset{˜}{b}}^{j}\Delta t+\left(I+{\overset{˜}{a}}_{1}^{j}\Delta t\right){\overset{˜}{R}}^{j}{\left(I+a_{1}^{j}\Delta t\right)}^{*}\right){\left(C^{j}\right)}^{*}, \end{array}$$

and the auxiliary matrix C is given by

(A20) $${\overset{˜}{C}}^{j}={\overset{˜}{R}}^{j}{\left(I+{\overset{˜}{a}}_{1}^{j}\Delta t\right)}^{*}{\left({\overset{˜}{b}}^{j}\circ {\overset{˜}{b}}^{j}\Delta t+\left(I+{\overset{˜}{a}}_{1}^{j}\Delta t\right){\overset{˜}{R}}^{j}{\left(I+{\overset{˜}{a}}_{1}^{j}\right)}^{*}\right)}^{-1}.$$

Note that the first equality in (A18) is due to the Markovian property of the underlying system [40]. In fact, if ${\overset{˜}{Y}}^{j+1}$ is known, then the conditional distribution of ${\overset{˜}{Y}}^{j}$ has no dependence on ${\overset{˜}{X}}^{s},s\geq j+1$. More specifically, the information of ${\overset{˜}{X}}^{s},s>j$ has been included in ${\overset{˜}{Y}}^{j+1}$. The conditional terms ${\overset{˜}{Y}}^{j+1}$ and ${\overset{˜}{X}}^{s},s\leq j$ represent the information in the future and past, respectively, which can be seen in (A19). This finishes the proof of Lemma A1.  □

The result in Lemma A1 allows the derivation of the conditional sampling formula. Each sampled point ${\overset{˜}{Y}}^{j}$ is conditioned on the previous one ${\overset{˜}{Y}}^{j+1}$ and the smoother estimate. The key step is to make use of the conditional probability in Lemma A1 to draw the sample and finally write down a SDE for an efficient calculation of the conditional sampled path. The proof is shown below.

**Proof.** Recall from (A18) that ${\overset{˜}{Y}}^{j+1}$ is calculated by generating a multivariate Gaussian random variable with mean ${\overset{˜}{m}}^{j}$ and covariance ${\overset{˜}{P}}^{j}$. The backward equation of sampling Y can be derived by making use of (A19) and (A20). Plugging (A20) into (A19b) yields

(A21) $$\begin{array}{cc} {\overset{˜}{P}}^{j} & ={\overset{˜}{R}}^{j}-{\overset{˜}{R}}^{j}{\left(I+{\overset{˜}{a}}_{1}^{j}\Delta t\right)}^{*}{\left({\overset{˜}{b}}^{j}\circ {\overset{˜}{b}}^{j}\Delta t+\left(I+{\overset{˜}{a}}_{1}^{j}\Delta t\right){\overset{˜}{R}}^{j}{\left(I+{\overset{˜}{a}}_{1}^{j}\Delta t\right)}^{*}\right)}^{-1}\left(I+{\overset{˜}{a}}_{1}^{j}\Delta t\right){\overset{˜}{R}}^{j}\\ \; & ={\overset{˜}{R}}^{j}-{\overset{˜}{R}}^{j}{\left(I+{\overset{˜}{a}}_{1}^{j}\Delta t\right)}^{*}{\left({\overset{˜}{R}}^{j}+\left({\overset{˜}{a}}_{1}^{j}{\overset{˜}{R}}^{j}+{\overset{˜}{R}}^{j}{\overset{˜}{a}}_{1}^{j}+{\overset{˜}{b}}^{j}\circ {\overset{˜}{b}}^{j}\right)\Delta t\right)}^{-1}\left(I+{\overset{˜}{a}}_{1}^{j}\Delta t\right){\overset{˜}{R}}^{j}+O\left(\Delta t^{2}\right)\\ \; & ={\overset{˜}{R}}^{j}-{\overset{˜}{R}}^{j}{\left(I+{\overset{˜}{a}}_{1}^{j}\Delta t\right)}^{*}{\left({\overset{˜}{R}}^{j}\left(I+{\left({\overset{˜}{R}}^{j}\right)}^{-1}\left({\overset{˜}{a}}_{1}^{j}{\overset{˜}{R}}^{j}+{\overset{˜}{R}}^{j}{\overset{˜}{a}}_{1}^{j}+{\overset{˜}{b}}^{j}\circ {\overset{˜}{b}}^{j}\right)\Delta t\right)\right)}^{-1}\left(I+{\overset{˜}{a}}_{1}^{j}\Delta t\right){\overset{˜}{R}}^{j}+O\left(\Delta t^{2}\right)\\ \; & ={\overset{˜}{R}}^{j}-{\overset{˜}{R}}^{j}{\left(I+{\overset{˜}{a}}_{1}^{j}\Delta t\right)}^{*}\left(I-{\left({\overset{˜}{R}}^{j}\right)}^{-1}\left({\overset{˜}{a}}_{1}^{j}{\overset{˜}{R}}^{j}+{\overset{˜}{R}}^{j}{\overset{˜}{a}}_{1}^{j}+{\overset{˜}{b}}^{j}\circ {\overset{˜}{b}}^{j}\right)\Delta t\right){\left({\overset{˜}{R}}^{j}\right)}^{-1}\left(I+{\overset{˜}{a}}_{1}^{j}\Delta t\right){\overset{˜}{R}}^{j}+O\left(\Delta t^{2}\right) \end{array}$$

For notation simplicity, we define

(A22) $$F={\left({\overset{˜}{R}}^{j}\right)}^{-1}\left({\overset{˜}{a}}_{1}^{j}{\overset{˜}{R}}^{j}+{\overset{˜}{R}}^{j}{\overset{˜}{a}}_{1}^{j}+{\overset{˜}{b}}^{j}\circ {\overset{˜}{b}}^{j}\right),$$

and thus

(A23) $$\begin{array}{cc} \; & {\overset{˜}{R}}^{j}{\left(I+{\overset{˜}{a}}_{1}^{j}\Delta t\right)}^{*}\left(I-{\left({\overset{˜}{R}}^{j}\right)}^{-1}\left({\overset{˜}{a}}_{1}^{j}{\overset{˜}{R}}^{j}+{\overset{˜}{R}}^{j}{\overset{˜}{a}}_{1}^{j}+{\overset{˜}{b}}^{j}\circ {\overset{˜}{b}}^{j}\right)\Delta t\right){\left({\overset{˜}{R}}^{j}\right)}^{-1}\left(I+{\overset{˜}{a}}_{1}^{j}\Delta t\right){\overset{˜}{R}}^{j}\\ = & {\overset{˜}{R}}^{j}{\left(I+{\overset{˜}{a}}_{1}^{j}\Delta t\right)}^{*}\left(I-F\Delta t\right){\left({\overset{˜}{R}}^{j}\right)}^{-1}\left(I+{\overset{˜}{a}}_{1}^{j}\Delta t\right){\overset{˜}{R}}^{j}\\ = & \left({\overset{˜}{R}}^{j}+{\overset{˜}{R}}^{j}{\overset{˜}{a}}_{1}^{j}\Delta t\right)\left({\left({\overset{˜}{R}}^{j}\right)}^{-1}-F{\left({\overset{˜}{R}}^{j}\right)}^{-1}\Delta t\right)\left({\overset{˜}{R}}^{j}+{\overset{˜}{a}}_{j}^{j}{\overset{˜}{R}}^{j}\Delta t\right)\\ = & \left(I-{\overset{˜}{R}}^{j}F{\left({\overset{˜}{R}}^{j}\right)}^{-1}+{\overset{˜}{R}}^{j}{\overset{˜}{a}}_{1}^{j}{\left({\overset{˜}{R}}^{j}\right)}^{-1}\Delta t\right)\left({\overset{˜}{R}}^{j}+{\overset{˜}{a}}_{1}^{j}{\overset{˜}{R}}^{j}\Delta t\right)\\ = & {\overset{˜}{R}}^{j}-{\overset{˜}{R}}^{j}F\Delta t+{\overset{˜}{R}}^{j}{\overset{˜}{a}}_{1}^{j}\Delta t+{\overset{˜}{a}}_{1}^{j}{\overset{˜}{R}}^{j}\Delta t+O\left(\Delta t^{2}\right). \end{array}$$

Plugging (A23) back to (A21) yields

(A24) $$\begin{array}{cc} {\overset{˜}{P}}^{j} & ={\overset{˜}{R}}^{j}-\left({\overset{˜}{R}}^{j}-{\overset{˜}{R}}^{j}F\Delta t+{\overset{˜}{R}}^{j}{\overset{˜}{a}}_{1}^{j}\Delta t+{\overset{˜}{a}}_{1}^{j}{\overset{˜}{R}}^{j}\Delta t\right)+O\left(\Delta t^{2}\right)\\ \; & ={\overset{˜}{R}}^{j}F\Delta t-{\overset{˜}{R}}^{j}{\overset{˜}{a}}_{1}^{j}\Delta t-{\overset{˜}{a}}_{1}^{j}{\overset{˜}{R}}^{j}\Delta t+O\left(\Delta t^{2}\right)\\ \; & ={\overset{˜}{b}}^{j}\circ {\overset{˜}{b}}^{j}\Delta t+O\left(\Delta t^{2}\right) \end{array}$$

Applying a similar argument, plugging (A20) into (A19a) yields and subtracting ${\overset{˜}{Y}}^{j+1}$ on both sides of (A19a) yields

(A25) $$\begin{array}{cc} {\overset{˜}{m}}^{j}-{\overset{˜}{Y}}^{j+1} & ={\overset{˜}{\mu }}^{j}+{\overset{˜}{R}}^{j}{\left(I+{\overset{˜}{a}}_{1}^{j}\Delta t\right)}^{*}{\left({\overset{˜}{b}}^{j}\circ {\overset{˜}{b}}^{j}\Delta t+\left(I+{\overset{˜}{a}}_{1}^{j}\Delta t\right){\overset{˜}{R}}^{j}{\left(I+{\overset{˜}{a}}_{1}^{j}\right)}^{*}\right)}^{-1}\\ \; & \;\;\;\times \left({\overset{˜}{Y}}^{j+1}-{\overset{˜}{a}}_{0}^{j}\Delta t-\left(I+{\overset{˜}{a}}_{1}^{j}\Delta t\right){\overset{˜}{\mu }}^{j}\right)-{\overset{˜}{Y}}^{j+1}\\ \; & ={\overset{˜}{\mu }}^{j}+{\overset{˜}{R}}^{j}{\left(I+{\overset{˜}{a}}_{1}^{j}\Delta t\right)}^{*}\left(I-{\left({\overset{˜}{R}}^{j}\right)}^{-1}\left({\overset{˜}{a}}_{1}^{j}{\overset{˜}{R}}^{j}+{\overset{˜}{R}}^{j}{\overset{˜}{a}}_{1}^{j}+{\overset{˜}{b}}^{j}\circ {\overset{˜}{b}}^{j}\right)\Delta t\right){\left({\overset{˜}{R}}^{j}\right)}^{-1}\\ \; & \;\;\;\times \left({\overset{˜}{Y}}^{j+1}-{\overset{˜}{a}}_{0}^{j}\Delta t-\left(I+{\overset{˜}{a}}_{1}^{j}\Delta t\right){\overset{˜}{\mu }}^{j}\right)-{\overset{˜}{Y}}^{j+1}+O\left(\Delta t^{2}\right)\\ \; & ={\overset{˜}{\mu }}^{j}+\left({\overset{˜}{R}}^{j}+{\overset{˜}{R}}^{j}{\overset{˜}{a}}_{1}^{j}\Delta t\right)\left({\left({\overset{˜}{R}}^{j}\right)}^{-1}-{\left({\overset{˜}{R}}^{j}\right)}^{-1}\left({\overset{˜}{a}}_{1}^{j}{\overset{˜}{R}}^{j}+{\overset{˜}{R}}^{j}{\overset{˜}{a}}_{1}^{j}+{\overset{˜}{b}}^{j}\circ {\overset{˜}{b}}^{j}\right){\left({\overset{˜}{R}}^{j}\right)}^{-1}\Delta t\right)\\ \; & \;\;\;\times \left({\overset{˜}{Y}}^{j+1}-{\overset{˜}{a}}_{0}^{j}\Delta t-\left(I+{\overset{˜}{a}}_{1}^{j}\Delta t\right){\overset{˜}{\mu }}^{j}\right)-{\overset{˜}{Y}}^{j+1}+O\left(\Delta t^{2}\right)\\ \; & ={\overset{˜}{\mu }}^{j}+\left(I-({\overset{˜}{a}}_{1}^{j}+{\overset{˜}{b}}^{j}\circ {\overset{˜}{b}}^{j})\Delta t\right)\left({\overset{˜}{Y}}^{j+1}-{\overset{˜}{\mu }}^{j}-\left({\overset{˜}{a}}_{0}^{j}+{\overset{˜}{a}}_{1}^{j}{\overset{˜}{\mu }}^{j}\right)\Delta t\right)-{\overset{˜}{Y}}^{j+1}+O\left(\Delta t^{2}\right)\\ \; & =-\left({\overset{˜}{a}}_{0}^{j}+{\overset{˜}{a}}_{1}^{j}{\overset{˜}{\mu }}^{j}\right)\Delta t-\left({\overset{˜}{a}}_{1}^{j}+{\overset{˜}{b}}^{j}\circ {\overset{˜}{b}}^{j}{\left({\overset{˜}{R}}^{j}\right)}^{-1}\right)\left({\overset{˜}{Y}}^{j+1}-{\overset{˜}{\mu }}^{j}\right)\Delta t+O\left(\Delta t^{2}\right)\\ \; & =-\left({\overset{˜}{a}}_{0}^{j}+{\overset{˜}{a}}_{1}^{j}{\overset{˜}{Y}}^{j+1}\right)\Delta t-{\overset{˜}{b}}^{j}\circ {\overset{˜}{b}}^{j}{\left({\overset{˜}{R}}^{j}\right)}^{-1}\left({\overset{˜}{Y}}^{j+1}-{\overset{˜}{\mu }}^{j}\right)\Delta t+O\left(\Delta t^{2}\right) \end{array}$$

Combining (A23) and (A25) and taking the limit Δt→0 yields an explicit formula of sampling the unobserved processes Z(t),

(A26) $$\overset{\leftarrow }{\;dY}=\left(-a_{0}-a_{1}Y\right)\;dt+\left(b\circ b\right)R^{-1}\left(\mu -Y\right)dt+{\left(b\circ b\right)}^{1/2}\;dW_{Y},$$

which is Corollary A1. Subtracting the smoother posterior mean from (A26) yields

(A27) $$\overset{\leftarrow }{\;dY}=\overset{\leftarrow }{\;d\mu_{s}}-\left(a_{1}+\left(b\circ b\right)R_{f}^{-1}\right)\left(Y-\mu_{s}\right)\;dt+{\left(b\circ b\right)}^{1/2}\;dW_{Y},$$

which is Theorem 3.  □
